# Life-space and movement behavior in nursing home residents: results of a new sensor-based assessment and associated factors

**DOI:** 10.1186/s12877-017-0430-7

**Published:** 2017-01-28

**Authors:** Carl-Philipp Jansen, Mona Diegelmann, Eva-Luisa Schnabel, Hans-Werner Wahl, Klaus Hauer

**Affiliations:** 1 Department of Psychological Aging Research, Institute of Psychology, Heidelberg University, Bergheimer Str. 20, 69115 Heidelberg, Germany; 2 Network Aging Research, Heidelberg University, Bergheimer Str. 20, 69115 Heidelberg, Germany; 3 Department of Geriatric Research, Agaplesion Bethanien Hospital, Geriatric Center at Heidelberg University, Rohrbacher Str. 149, 69126 Heidelberg, Germany

**Keywords:** Life-space, Nursing home, Sensor-based assessment, Spatiotemporal movement behavior

## Abstract

**Background:**

Studies on life-space (LS) and its determinants have previously been limited to community-dwelling subjects but are lacking in institutionalized older persons. The purpose of this study was to provide an advanced descriptive analysis of LS in nursing home residents and to identify associated factors based on an established theoretical framework, using an objective, sensor-based assessment with a high spatiotemporal resolution.

**Methods:**

Cross-sectional study in two nursing homes in Heidelberg, Germany (*n* = 65; mean age: 82.9 years; 2/3 female). Changes of location in the nursing home (Transits) as well as time spent away from the private room (TAFR) were assessed using a wireless sensor network. Measures of physical, psychosocial, cognitive, socio-demographic, and environmental factors were assessed via established motor performance tests, interviews, and proxy-reports.

**Results:**

LS of residents was largely restricted to the private room and the surrounding living unit (90%); 10% of daytime was spent outside the living unit and/or the facility. On average, TAFR was 5.1 h per day (±2.3; Range: 0–8); seven Transits (6.9 ± 3.2; Range: 0–18) were performed per day. Linear regression analyses revealed being male, lower gait speed, higher cognitive status, and lower apathy to be associated with more Transits; higher gait speed, lower cognitive status, and less depressive symptoms were associated with more TAFR. LS was significantly increased during institutional routines (mealtimes) as compared to the rest of the day.

**Conclusions:**

The sensor-based LS assessment provided new, objective insights into LS of institutionalized persons living in nursing homes. It revealed that residents’ LS was severely limited to private rooms and adjacent living units, and that in institutional settings, daily routines such as meal times seem to be the major determinant of LS utilization. Gait speed, apathy, and depressive symptoms as well as institutional meal routines were the only modifiable predictors of Transits and/or TAFR, and thus have greatest potential to lead to an enhancement of LS when targeted with interventions.

**Trial registration:**

Current Controlled Trials ISRCTN96090441 (retrospectively registered).

## Background

Considerable efforts have been made to increase quality of care and quality of life in nursing home residents (NHR) in the past decades [1, 2], but physical and social inactivity still remain a large concern in modern nursing facilities. As research has shown, the majority of NHR spend their time inactively sitting or lying alone [3, 4]. A measure that has been positively associated with physical activity as well as social participation in community-dwelling older adults [5, 6] and NHR [7] is life-space (LS). LS has been conceptualized as the spatial extension of an individual’s environment that he/she moves in during a specified time period [5, 8], irrespective of the types of conducted activity, use of walking aids, or other assistance [9]. In general, greater LS implies that an individual has more opportunity to visit personally meaningful places and to interact socially with others [10]. To highlight the complexity of factors influencing LS in older adults, Webber et al. [11] presented a theoretical framework in which LS mobility in old age is assumed to be influenced by cognitive, psychosocial, physical, socio-demographic, financial, and environmental dimensions. The model has found partial empirical support in previous research which has demonstrated associations between LS mobility and physical performance [8, 12, 13], global cognitive functioning [14], and psycho-social factors including depression [12, 13, 15], concerns about falling [16], and apathy [13] in community-dwelling older persons.

Applying the LS framework in NHR—an institutionalized group of older persons with multiple impairments—poses the question whether the determinants identified in community-dwelling older subjects can also be verified for LS in NHR. Institutions such as nursing homes (NH) are expected to have pronounced characteristics that strongly determine life and behavior within them [17]. Such characteristics are, for example, architectural features (e.g., special care units, meeting places); care routines; or institution-dependent organizational schedules including meal times and weekly recurring and highly standardized events [18, 19]. That said, we assume that the framework of Webber et al. (2010) may largely be valid also for the NH setting, but needs additional qualification in that the institutional factors impacting on behavior may play a key role in determining LS.

LS has mostly been assessed in- and outside of the private home environment [8, 9, 12, 20]. Self-report measurements as the *Life-Space Diary* [8], the *University of Alabama at Birmingham Study of Aging Life-Space Assessment* [12], or the *Life-Space Questionnaire* [9] were predominantly used, providing a composite score of LS across a defined time period. Regarding NHR, only one measure has been introduced to our knowledge, a proxy-rating titled *Nursing Home Life-Space Diameter* (NHLSD) [19]. Such subjective LS assessments—self- or proxy-ratings—come with multiple weaknesses, e.g., recall/response biases, especially in cognitively impaired subjects [21–23]. Moreover, they are unable to identify changes or events with temporal precision and intra-individual specificity [24]. Also, predictors of LS may operate differently in more global questionnaire data as compared to high resolution data. This is why increasingly objective, technical LS-related assessments [13, 20, 25] (e.g., Global Positioning System, infrared motion sensors, or Bluetooth transmitters) are used which also provide a high spatiotemporal resolution of LS not achievable by questionnaire-based assessment. However, such an advanced assessment strategy has so far not been applied in the nursing home setting. Specifically, a continuous, real-time assessment with high spatiotemporal resolution and minimal intrusion of the daily activities of individuals allows a more accurate picture of LS dynamics in daily ecologies and thus higher ecological validity. It may also become important as an endpoint in intervention research or serve diagnostic purposes by adding information to clinical status assessments.

This study provides a new sensor-based LS assessment in an institutional setting, including automated, high-resolution, spatiotemporal recording of residents’ habitual movement behavior within the resident facility across daytime. The aim of this study is threefold: (1) We provide a highly accurate picture of NHR’ LS and movement behavior, not achievable by previously used assessments. (2) Based on the theoretical LS framework of Webber et al. (2010), we examine whether its LS determinants are applicable to the nursing home setting and allow to develop a model explaining variance in NHR’ LS. (3) Given the unique characteristics of institutions described above, we hypothesize that, in addition to the factors described by Webber et al., a large proportion of the variance in NHR’ LS will be attributable to institutional routines, i.e., scheduled mealtimes.

## Methods

### Design

The present study is based on cross-sectional data from Long-Term Care in Motion (LTCMo, ISRCTN96090441, [26]). Ethical approval for the project was obtained from the Ethic Review Board of the Faculty of Behavioral and Cultural Studies at Heidelberg University. The study was conforming to the respective policy and mandates of the Declaration of Helsinki. Either residents or their legal representative provided written informed consent.

### Participants

Participant characteristics are presented in Table 1. Participants were permanent residents of two comparable nursing homes in Heidelberg, Germany, that were situated in a quiet, suburban residential area, with promenades and supermarkets close by. The surrounding area was easy to access and did not include mobility barriers such as busy streets or hills. Both homes were obliged to newest care standards, run by the same organization and equal in organizational structures, neighborhood, mealtimes, and activity programs. Their architectural conceptualization was equal, which makes both facilities comparable. Both facilities had long hallways along which the private rooms where located; hallways all met in a large public area where meetings and group activities took place and meals were served; easily accessible elevators and stairs allowed transfer to other units on different building levels. Except for those who were terminally ill or received palliative care, all residents were eligible for participation. Of 259 permanent residents in both nursing homes, 137 gave consent to participating in the LS assessment, of which 65 fully completed both measurement days. Reasons for exclusion from analysis were removal of sensors (*n* = 13) or incomplete data due to technical difficulties identified via maintenance software running alongside measurement, i.e., reception disturbance or damaging of hardware by NHR (*n* = 36); measurement interruption due to a power breakdown one morning (*n* = 21); and measurement inaccuracy, i.e., the system could not distinguish between zone 1 and 2 in residents having their private room next to the dining area (*n* = 2).

**Table 1:** Participant Characteristics and Descriptive Statistics on LS Data

	*N*	Mean (SD)	Range
Age [years]	65	82.9 (9.6)	53–98
Sex [female / male]	43 / 22		
Length of stay [years]	65	2.2 (1.7)	0–8
Nursing Home 1 / 2	27 / 38		
Open / code-secured unit	53 / 12		
MMSE [score]	58	18.0 (8.1)	2–30
AES-D [score]	65	15.4 (8.7)	0–28
GDS-12R [score]	56	3.0 (3.3)	0–11
FES-I [score]	55	9.5 (3.2)	7–21
Max. gait speed [m/s]	61	0.57 (0.50)	0–1.99
Ambulatory status walk without aid walk with aid wc, self-propelled wc, immobile	65 15 29 10 11		
Time spent in Z1 [h]	65	2.93 (2.33)	0–8
Time spent in Z2 [h]	65	4.30 (2.39)	0–8
Time spent in Z3 [h]	65	0.47 (0.61)	0–2.24
Time spent in Z4 [h]	65	0.31 (0.81)	0–4.13
TAFR [h]	65	5.07 (2.33)	0–8
Transits [n]	65	6.9 (3.2)	0–18

*Abbreviations*: *AES-D* Apathy Evaluation Scale, *FES-I* Falls Efficacy Scale International, *GDS-12R* Geriatric Depression Scale-Residential, *[h]* hours, *MMSE* Mini-Mental State Examination, *[m/s]* meters per second, *[n]* number, *SD* standard deviation, *TAFR* time spent away from private room, *wc* wheelchair, *Z* Zone

### Data collection

**Fig. 1:**
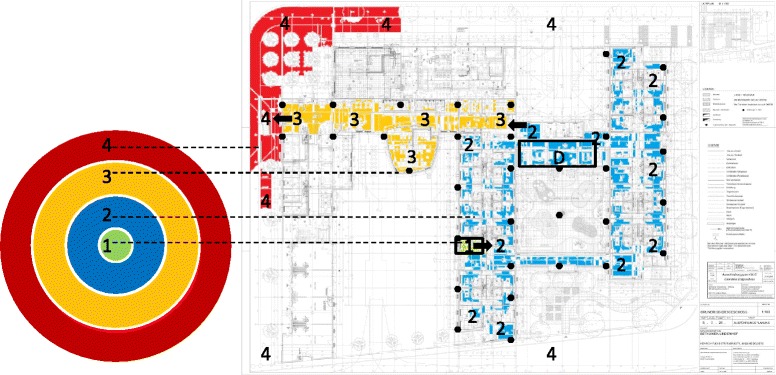
Overview of one nursing home including the division of life-space into four hierarchical zones. Hierarchical zones are delineated as concentric circles as defined by Tinetti & Ginter (1990). *Black dots* indicate positions of wireless receivers of the measurement system, encasing the whole building; Zone 1 (*green*) stands for the private room; Zone 2 (*blue*) stands for the whole living unit in which the private room is located; Zone 3 (*orange*) stands for the public area outside the living unit but within the facility; Zone 4 (*red*) delineates the whole area outside the facility. *Arrows point* out passages between zones. Zone 1 (exemplary private room) is bordered with a *black frame*. The dining area within Zone 2 is marked with a “D” and bordered with another *black frame*

In order to achieve an advanced descriptive analysis of LS, s-net® technology (Fraunhofer Institute for Integrated Circuits IIS, Erlangen, Germany [27, 28]) was used in both nursing homes. This technology uses mobile nodes (end nodes) that determine their position at 30 s intervals based on Received Signal Strength Indicator (RSSI) values. RSSI values were calculated based on received messages from wireless communication. An infrastructure of anchor nodes was built within the building as position references for the network’s routing function. The anchor nodes were evenly distributed on outside walls at height of approximately 2.3 m in both nursing homes, spanning a polygonal area within which end nodes could determine their position (Fig. 1). To ensure identical density of anchor node distribution in both facilities, 72 anchor nodes were distributed in nursing home 1 and 151 anchor nodes in nursing home 2, due to the different size of the buildings. Anchor nodes were configured with fixed positions that were broadcasted periodically during measurement, operating on a transmission frequency of 868 MHz. End nodes being within broadcast range of anchor nodes received these messages and measured their signal strength. From three to 16 received reference positions of anchor nodes a weighted centroid was calculated, being defined as the estimate of the end node’s position. The weights were derived from the measured RSSI values such that the calculated position was closer to the anchor nodes with higher signal strength. The calculations were based on the underlying algorithm (Weighted Centroid Location; WCL [29]). RSSI measurements can be severely affected by multipath fading and shadowing on end nodes, resulting in fluctuations of measured values. To address this issue, s-net® localization contains filter components for pre- and post-processing of measured values. Validation of the system showed a mean deviation of 2.28 m (range: 0.3–4.6 m) of the end nodes’ physical position [28].

After the system components had been installed in the nursing homes, the network’s connectivity was tested and warranted before measurement started. During the first four measurement days, a Fraunhofer technician monitored communication between sensors using maintenance software that could immediately identify technical problems or failure of single sensors. Due to network capacities, a maximum of 22 participants at a time were equipped with sensors for two consecutive days; the average of both days was used for the analyses. Residents were visited on normal weekdays and equipped with one end node each morning as soon as they left their private room and entered the public dining room. They kept the end node until they returned to their private room in the evening after dinner. To achieve comparability between subjects, LS data were analyzed for each participant from 10 a.m. to 6 p.m. (hereafter referred to as “daytime”).

Several steps were taken to control for actual end node wearing time. During measurement, participants were visited every two to three hours to ensure adherence to measurement protocol. In addition, nursing staff were asked to immediately report on lost/found end nodes in order to follow up on reasons for loss or to continue measurement if appropriate. If position reports of end nodes were not received steadily according to maintenance software, end nodes were immediately checked. If participants had not worn end nodes constantly during measurement, they were excluded from analysis. In case of occasional gaps where end nodes were without reception within the network, the duration of these gaps was added to the duration of the preceding episode. This was based on the assumption that an end node is more likely to regain reception as soon as the person wearing it changes her/his location.

Based on previous research [19], the nursing home life space was hierarchically structured into four zones (Fig. 1): private room (Zone 1); outside the room but within the living unit (Zone 2); outside the living unit but within the facility (Zone 3); outside the facility (Zone 4).

Using a previous analytic concept to operationalize LS in community-dwelling persons [20], two LS-parameters were derived from LS raw data to describe relevant behavioral features of residents’ spatiotemporal movement in the nursing home environment: the time residents spent away from their private room (TAFR) and the frequency of LS zone changes (Transits).

- *Cognitive Factors*. Cognitive Status was assessed using the *Mini-Mental State Examination* (MMSE) [30].
- *Psychosocial Factors*. Psychological status was assessed by established assessment methods validated in persons with cognitive impairment or in NHR (12-item *Geriatric Depression Scale—Residential* (GDS-12R) for depression [31], *Apathy Evaluation Scale* (AES-D) [32–34] for apathy, and the *Short Falls Efficacy Scale International* (Short FES-I) [35, 36] for fall-related self-efficacy [37].
- *Environmental Factors*. TAFR and Transits during institutionally scheduled mealtimes*—*including 15 min transfer time before and after*—*were extracted to operationalize institutional routines. Unscheduled LS was defined as TAFR and Transits during the rest of the daytime. In total, institutionally scheduled mealtimes constitute 2.7 h of the overall measured daytime (8.0 h).
- *Physical Factors*. Based on observations and staff information, residents were rated regarding their *ambulatory status* as *(a)* ambulatory without aid, *(b)* ambulatory with aid, *(c)* self-propelled wheelchair user, and *(d)* fully immobile wheelchair user. Gait speed was assessed with a *10 m walk test* at maximum walking speed, using a walking aid if necessary.
- *Socio-Demographic Factors*. Age, sex, and length of stay in the facility were assessed using the care documentation.

### Data analysis

Descriptive LS analysis included mean, standard deviation, and range for all variables. Relative strength of associations between LS measures (TAFR and Transits; average of both measurement days) and independent variables were determined by linear regression models. Variables were considered for inclusion into the regression models based on structural coherence with the dimensions described in the LS mobility framework by Webber et al. (2010), except for financial factors which, as we assume, play no role in our sample. Given our rather small sample size, predictors that were not correlated with the criterion variables (bivariate correlations of either *r* or *rho* < .2 and *p* > .10; Table 2) were not included in the models; only one factor was included for each dimension. To avoid multicollinearity in case of several factors of the same domain being correlated to dependent variables, these were included separately in the regression models and the strongest factor was then selected. To explore the association between institutional routines and LS, we repeated the regression analysis but controlled for the variance of Transits and TAFR during institutionally scheduled mealtimes. Regressions were based on full information maximum-likelihood (FIML) estimations which consider all available data from all respondents, thus avoiding selective case deletion and maintaining sample size-dependent power. FIML provides unbiased estimations given that data is missing at random and multivariate normal [38]. To account for non-normality, we used a robust maximum likelihood estimator. Dependent t-tests for paired samples were computed to analyze differences between LS during institutionally scheduled mealtimes and unscheduled daytime. All statistical analyses were performed using SPSS for Windows (IBM SPSS Statistics for Windows, Version 23.0. Armonk, NY: IBM Corp.) and Mplus version 7.31 [39].

**Table 2:** Bivariate Correlations Between LS-Measures and Predictor Variables

	TAFR	Transits
Age [years]	−.10	−.15
Sex	−.07^‡^	.36**^‡^
Length of Stay [years]	.01	.08
MMSE [score]	−.47***	.34**
Gait Speed [m/s]	.51***	−.28^+^
GDS-12R [score]	−.35**	.15
FES-I [score]	−.36**	.28*
AES-D [score]	.09	−.28*
Ambulatory Status	.08^‡^	.13^‡^

*Abbreviations*: *AES-D* Apathy Evaluation Scale, *FES-I* Falls Efficacy Scale International, *GDS-12R* Geriatric Depression Scale-Residential, *MMSE* Mini-Mental State Examination, *TAFR* time spent away from private room, *wc* wheelchair, *yrs* years, *†* Pearson r, *‡* Spearman rho

^+^ = <.10; * = *p* < .05; ** = *p* < .01; *** = *p* ≤ .001

## Results

### Descriptive life-space statistics

According to the s-net® measurement protocol, approximately 62,400 position reports were received during the study, which is equal to 480 observations of each NHR’s position per day [62,400 / (65 NHR × 2 days)]. Results of the LS measures TAFR, Transits, and the average duration of stay in the four LS-zones (displayed in Table 1) on both days show that LS of residents was to a very large extent restricted to the private room (zone 1: 2.93 h = 36.6% of the daytime) and the immediate area around it (zone 2: 4,30 h = 53,8%). On average, NHR spent only 0.47 h (=5.9%) outside the own unit but within the facility (zone 3) and only 0.31 h (=3.8%) outside the facility (zone 4) per day. Three quarters of the residents went beyond their living unit and one quarter left the facility at least once during both measurement days. On average, almost seven Transits (6.9 ± 3.2; Range: 0–18) were made. Only two residents (3.1%) never left their room whereas 22 residents (33.8%) spent less than one hour in their room during daytime. Intraclass Correlation Coefficients (ICCs) showed fair agreement between both measurement days for Transits (.41) and strong agreement for TAFR (.76).

**Fig. 2:**
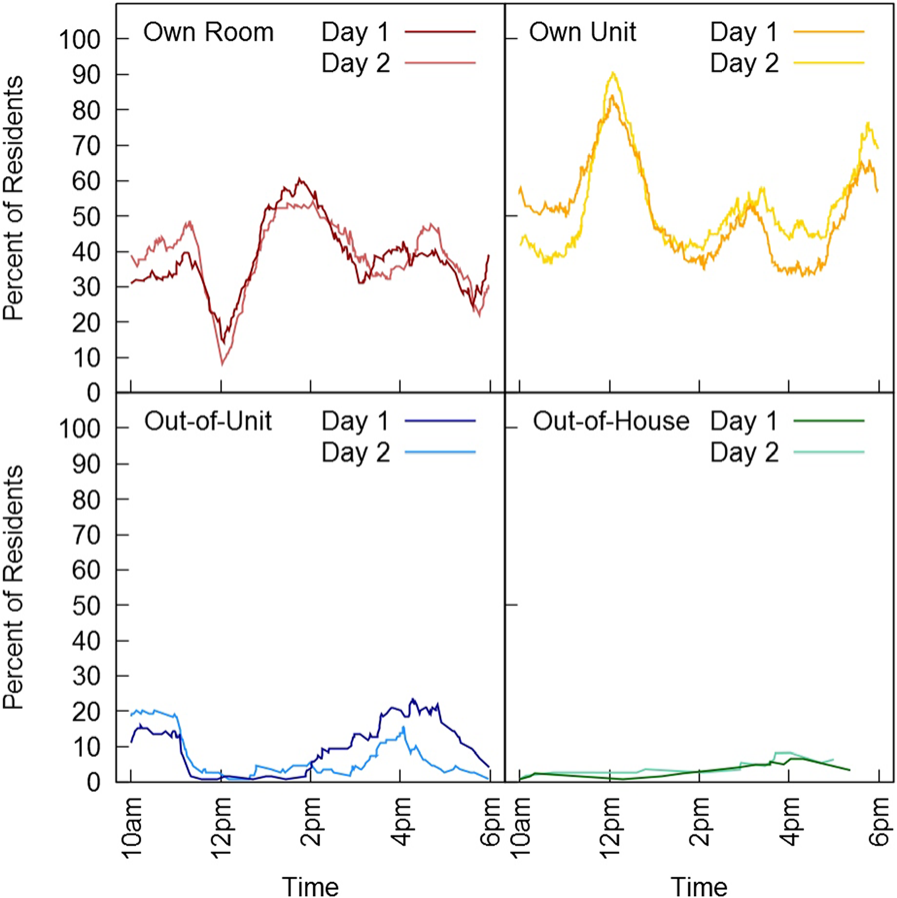
Percentage of NHR in each of the life-space zones across two measurement days. Most resident Transits occurred during lunch time between 11.30 a.m. and 1.00 p.m. and during dinner time around 5.20 p.m. and 6.00 p.m. The only time frames in which several residents left their own living unit were between 10 a.m. and lunch and between 2 p.m. and dinner (see Fig. 2). The highest number of residents (8%) was located outside the facility around 4 p.m

Figure 2 shows residents’ LS with spatial *and* temporal resolution, i.e., the percentage of residents measured in each LS zone across the daytime.

### Predictors of life-space in NHR

Results of bivariate correlation analysis are presented in Table 2; results of linear regression analyses in Table 3. Male sex, lower gait speed, lower apathy, and higher cognitive status were associated with higher amounts of Transits and jointly accounted for 27% of the variance (*p* = .002). Apathy and cognitive status, however, were not significant in the regression model.

**Table 3:** Linear Regression Analyses—Models for Transits and TAFR

	β	SE	Sβ
Transits			
Sex	3.02**	1.03	.34**
Gait Speed	−2.27*	1.14	−.21*
AES-D	−.10	.07	−.21
MMSE	.07	1.14	.13
R^2^			.27**
TAFR			
Gait Speed	2.34***	.61	.38***
MMSE	−.10***	.03	−.36***
GDS-12R	−.14*	.07	−.20*
R^2^			.43***

*Abbreviations*: *β* raw β, *R* ^*2*^ overall R^2^ of each model, *Sβ* standardized β, *SE* standard error, *AES-D* Apathy Evaluation Scale, *GDS-12R* Geriatric Depression Scale-Residential, *MMSE* Mini-Mental State Examination, *TAFR* time spent away from private room

^+^ *p* < .10; * *p* < 0.05; **p < 0.01; ***p < 0.001

In the model for TAFR, higher gait speed, lower cognitive status and less depressive symptoms were significantly associated with more TAFR. The model accounted for 43% of the variance (*p* < .001). Although concerns about falling were significantly correlated with TAFR, Short FES-I scores were not included in the final model as they explained less of the variance than GDS scores and did not contribute more to the overall variance explanation of the model.

### LS and institutional routines

When subsequently included in the models, TAFR and Transits during institutionally scheduled mealtimes showed a very strong effect on overall TAFR and Transits. TAFR during these explained almost 80% of the variance of the overall TAFR (*R* ^2^ = .80, β = 2.83; *p* < .001), leaving all other predictors insignificant. A similar effect was observed for Transits (*R* ^2^ = .67, β = 1.78; *p* < .001). During institutionally scheduled mealtimes, residents spent significantly more TAFR per hour than during unscheduled daytime (70.0% vs. 59.8%, *t* = 4.24, *p* < .001) and performed more Transits per hour (1.58 vs. 0.69, *t* = 11.35, *p* < .001).

## Discussion

To the best of our knowledge, this is the first study to explore LS in NHR based on objective, sensor-based assessment with a high spatiotemporal resolution. Key findings of the current study were that (1) LS was very limited in NHR; (2) factors belonging to dimensions included in the framework by Webber et al. (2010) are also applicable to the NH setting; (3) when included in the models, the strongest association was found between overall LS variables and institutionally scheduled routines such as mealtimes.

Following the methodological paths of key studies in the field of LS research [8, 9, 12, 19], we took a different practical approach by using a sensor-based system to obtain a comprehensive and objective picture of LS in NHR. Unlike subjective assessments used in previous studies, our objective assessment approach is not limited to generating a composite LS score, but also provides data on the chronological order in which LS areas where visited and for how long. This allows investigating LS far more extensively than before, including aspects of daily movement behavior in a sample of highly vulnerable NHR with high prevalence of advanced motor and cognitive impairment.

Despite the rather tight corset of the daily structure in institutions like nursing homes, data analysis revealed a wide spectrum of LS, ranging from residents who permanently stayed in their private room to those who were permanently absent from their room during daytime (see Table 1). Due to the lack of LS-related research in the nursing home setting, there are no results available for comparison with our findings on the duration a subject spent at a certain room or the frequency in which s/he changed zones on a daily basis in NHR. Results from studies using the NHLSD [7, 19] are hardly comparable as this measure is conceptualized as composite LS score regarding the past 2 weeks. When compared to independent-living seniors, NHR perform considerably less Transits (6.9 per day vs. 10.8 room changes per hour) and spent muss less time out of the house (0.3 h per day vs. 4.0 h per day) [20]. It has to be taken into account that in these two groups, the different LS zones have a different connotation, e.g., TAFR for a NHR still means staying indoors, whereas time away from home for an independent-living subject means leaving the building.

Our data shows that the main part of NHR’ daily life unfolded on the living units. Only very few individuals left their unit or facility and thus were not engaged in any activity beyond the facility at all. This is in line with Goffman (1961), who pointed out that institutions such as nursing homes are characterized by a “barrier to social intercourse with the outside” (p. 4). Some NHR may be worried by the thought of entering a less controlled, rather unknown and unsafe area beyond their unit. Others may feel drawn out of isolation in their room towards more eventful places. As a result, most NHR mainly stay in the public areas of their living units—a behavior which may also be attributable to motor and cognitive impairment of NHR.

Results from linear regression analyses confirm the LS-related dimensions identified by Webber et al. (2010) in their framework as well as findings in previous studies [8, 12–16]. In line with previous studies that found male sex being associated with larger LS mobility [40, 41], male sex was associated with more Transits in our sample.

Regarding motor performance, we found conflicting results on the association between LS and gait speed. Whereas more TAFR was associated with higher gait speed, more Transits were associated with lower gait speed. Thus, residents with better walking abilities change LS areas less frequently but stay in zones 2 to 4 for longer periods of time. One explanation may be that NHR with inferior walking abilities and functional capacity need to take rest periods in their private rooms more frequently than those with better physical function. We see this contrasting association of functional performance with both LS parameters as an indicator of different underlying concepts of both parameters requiring further investigation.

Our finding that lower cognitive status and less depressive symptoms were associated with TAFR finds support in results on community-dwelling subjects regarding the time out of home [20, 42]. Lower cognitive status was associated with more TAFR and less Transits in our sample. Cognitively impaired subjects probably feel drawn to public areas due to certain aspects of these areas that draw attention (e.g., noise or conversation) [43]. Due to diminished wayfinding abilities (i.e., not finding their own private room and staying where they presently are instead), or a high prevalence of apathy, they are often bound to stay in such public areas or other already determined locations. Cognitive performance may also reflect staffs’ reaction to these symptoms of dementia, that is, to keep residents in sight in the public area, and thus a larger amount of time is spent in public areas away from the own room, and less Transits are performed [44]. However, beta weights were not significant for MMSE scores in the Transits regression model. The same applies to beta weights for apathy, with lower apathy being associated with more Transits, as previously reported in community-dwelling subjects [13]. To explore this insignificance, we examined these linear regression results more closely. We found that AES-D scores were significantly correlated with MMSE scores (Pearson’s *r* = −.558; *p* < .001). When eliminating one of both measures from the regression model, the other factor became significant (AES-D: standardized beta = −.280; *p* = .005; MMSE: standardized beta = .253; *p* = .045), indicating that AES-D and MMSE have a considerable proportion of shared variance explanation. This is not surprising as apathy is a key symptom of dementia [45].

As expected, the strong association between institutional factors and LS became very clear. When controlling for the variance of Transits and TAFR during institutionally scheduled mealtimes in separate models, it explained 67% (Transits) and 80% (TAFR) of the variance of Transits and TAFR during overall daytime, even though it only stood for one third of the overall measurement time. In the presence of these control variables, all other predictors included in the final models became insignificant, which demonstrates the high association between the variance of LS parameters during mealtimes and overall variance, adding valuable information regarding the structure of the Webber et al. framework when applied in the nursing home setting. That is, when comparing institutionally scheduled mealtimes with unscheduled daytime, considerable differences in LS parameters were found. During institutionally scheduled mealtimes, there were twice as many Transits per hour and TAFR was more than 20% higher. This has several implications: First, institutionally scheduled time is a rather “active” time, as it requires the majority of otherwise rather sedentary residents to move (or be moved) to the dining area and to be around others in a social context. Compared to this active time, NHR actually tend to be less active when they can freely decide what to do, e.g., participate in optional social group activities. Second, it implies a restriction of LS in terms of its *range*, as NHR have to be *inside* and *within* the living units during these institutionally scheduled mealtimes if they want to be served their meal—unless they are invited and picked up for a meal by friends or relatives.

Several limitations of the study have to be noted. Although the sensor-based assessment provides an objective documentation of LS, this technological approach comes with some technical limitations, especially regarding gaps in data transmission. Due to the systems localization frequency of one per 30 s, Transits within this time frame could have been missed if more than one had occurred. However, due to the low gait speed and motor function of our sample, more than one Transit within 30 s is a rather unlikely event. As a relatively high number of participants had to be excluded from analyses, the study sample was limited and thus potentially underpowered for certain research questions. Some of the independent variables are based on self-report measures, which may have been affected by recall and response bias due to cognitive impairment or other factors such as depressive symptoms. The study design was intentionally inclusive, also including a minor group of persons being unable to move independently. However, we see LS as an objective reality, irrespective of its active or passive occurrence.

Some valuable practical implications arose from our study. With a view to the associated factors found in this study, and by identifying the individual movement patterns of each resident during the day, our assessment approach may also be suitable for documenting deterioration in motor function and development of depression or behavioral symptoms related to dementia such as apathy (manifesting as ‘never leaving the private room’) or wandering/restlessness (manifesting as ‘moving around constantly’). The fact that NHR are particularly inactive between meals shows the good occasion in the daily schedule for implementing physical activity and LS enhancing interventions. These should be focused on associated factors that are susceptible to intervention (especially gait, apathy, and depressive symptoms), and be carried out on the living units in order to be within reach of the majority of residents who do not go beyond their living unit. Overall, the sensor-based LS assessment is a good example of how new assessment strategies may provide new and more comprehensive insights into the movement behavior of NHR. As it is still undergoing further development, the sensor-based LS assessment promises to capture more complex parameters that may be derived from raw data in the future, e.g., distance travelled within the facility as a measure of physical activity. Our approach may also help in identifying architectural and environmental characteristics of NHs such as dangerous, fall-provoking spots or important meeting places, and unfrequented, deserted areas in the facility, allowing enrichment of the environment and further stimulation of NHR’ social participation in daily life.

## Conclusions

As derived from a sensor-based measurement for indoor localization, the LS of NHR was mainly limited to private rooms and living units. The LS framework by Webber et al. has proven useful in the NH setting as LS was associated with predictors similar to those previously identified in studies with community-dwelling subjects. However, it requires modification in that daily routines such as meal times should be included as a determinant in institutional settings due to their high impact on residents’ LS as revealed in regression models. Gait speed, apathy, and depressive symptoms as well as institutional meal routines were the only modifiable predictors of Transits and/or TAFR, and thus have the potential to lead to an enhancement of NHR LS and movement behavior when targeted with interventions.

## Abbreviations

[m/s]: Meters per second
AES-D: Apathy Evaluation Scale
FES-I: Falls Efficacy Scale International
FIML: Full Information Maximum-Likelihood
GDS-12R: Geriatric Depression Scale-Residential
LS: Life-space
MMSE: Mini-Mental State Examination
n: Group size
NH: Nursing home
NHLSD: Nursing Home Life Space Diameter
NHR: Nursing home residents
p: Significance level
r: Pearson’s r
rho: Spearman’s rho
RSSI: Received Signal Strength Indicator
SD: Standard deviation
Sβ: Standardized beta weight
TAFR: Time spent away from the private room
wc: Wheelchair
yrs: Years
β: Beta weight
